# Deciphering the role of host-gut microbiota crosstalk via diverse sources of extracellular vesicles in colorectal cancer

**DOI:** 10.1186/s10020-024-00976-8

**Published:** 2024-11-05

**Authors:** Yun Song, Min Shi, Yugang Wang

**Affiliations:** 1 Department of Gastroenterology, Tongren Hospital, Shanghai Jiao Tong University School of Medicine, 1111 Xianxia Road, Shanghai, 200336 PR China; 2 Key Laboratory for Translational Research and Innovative Therapeutics of Gastrointestinal Oncology, Tongren Hospital, Shanghai Jiao Tong University School of Medicine, Shanghai, 200336 PR China

**Keywords:** Extracellular vesicles, Exosomes, Colorectal neoplasms, Gastrointestinal microbiome, Probiotics

## Abstract

Colorectal cancer is the most common type of cancer in the digestive system and poses a major threat to human health. The gut microbiota has been found to be a key factor influencing the development of colorectal cancer. Extracellular vesicles are important mediators of intercellular communication. Not only do they regulate life activities within the same individual, but they have also been found in recent years to be important mediators of communication between different species, such as the gut microbiota and the host. Their preventive, diagnostic, and therapeutic value in colorectal cancer is being explored. The aim of this review is to provide insights into the complex interactions between host and gut microbiota, particularly those mediated through extracellular vesicles, and how these interactions affect colorectal cancer development. In addition, the potential of extracellular vesicles from various body fluids as biomarkers was evaluated. Finally, we discuss the potential, challenges, and future research directions of extracellular vesicles in their application to colorectal cancer. Overall, extracellular vesicles have great potential for application in medical processes related to colorectal cancer, but their isolation and characterization techniques, intercellular communication mechanisms, and the effectiveness of their clinical application require further research and exploration.

## Introduction

As the most common type of cancer in the digestive system, colorectal cancer (CRC) poses a significant threat to human health (Siegel et al. 2023a, b). Important risk factors include genetic mutations, racial and geographic differences, smoking, processed meat, alcohol use, obesity, adult height, and inflammatory bowel disease, encompassing both innate and acquired factors (Siegel et al. 2023a, b; Murphy and Zaki 2023). Recent studies have identified gut microbiota as another key factor influencing the development and progression of CRC (White and Sears 2023; Wong and Yu 2023). Specifically, pathogenic gut microbiota can promote the occurrence and development of CRC by producing genotoxins to promote the emergence of gene mutations, regulating oncogenic signal cascades, inducing colonic inflammation, promoting immunosuppression, and producing harmful metabolites such as hydrogen sulfide. On the other hand, various probiotics can inhibit the development of CRC by inhibiting the viability of CRC cells, inhibiting inflammation, reversing gut microbiota imbalance and promoting immune responses (Wong and Yu 2023). The definition of gut microbiota imbalance described in this review refers to the consensus statement published by the International Cancer Microbiome Consortium in 2019 (Scott et al. 2019). It is believed that dysbiosis is both disease-specific and host-specific. From the perspective of cancer, the microbiota should have a tumor-suppress effect on the host. Gut microbiota imbalance is regarded as a continuous deviation of the host microbiota from health-related homeostasis and a shift towards promoting or maintaining a cancerous phenotype. The gut microbiota, predominantly composed of various bacteria (Cho and Blaser 2012; Lynch and Pedersen 2016), interacts with the host through proteins (Sberro et al. 2019; Vidal-Veuthey et al. 2022; Kasarello et al. 2023; Duncan et al. 2021), nucleic acids (Liu et al. 2016; Li et al. 2020), metabolites (Cani 2016), and neurotransmitters (Kasarello et al. 2023). Similarly, the host can influence the composition of the gut microbiota and modulate physiological processes like immunity through the intake of exogenous food (Zhang 2022; Nova et al. 2022), drugs (Li et al. 2023a, b), and probiotics (Wang et al. 2021; Sanders 2011).

Extracellular vesicles (EVs) have recently gained attention as critical mediators in the emerging discovery of communication between gut microbiota and the host. Discovered in 1946 and initially thought to be a method of waste excretion (Chargaff and West 1946; Johnstone et al. 1991), EVs are now recognized as key intercellular communication mediators (Petroni et al. 2023; Liu and Wang 2023), which are being explored as biomarkers and drug carriers in the diagnosis and treatment of various diseases. Specifically, by detecting the content of specific molecules in EVs, the occurrence and progression of diseases can be predicted. EVs can be used for disease treatment by engineering EVs, loading drugs into cells that secrete EVs or directly loading drugs into EVs. For example, in vitro experiments have confirmed that SMAD3 promotes the adhesion of hepatocellular carcinoma cells. By adding SMAD inhibitors, the expression of various adhesion-related molecules can be blocked. It was found that there is abundant SMAD3 in the peripheral blood EVs of patients with hepatocellular carcinoma. Its content is positively correlated with disease stage and pathological grade, and negatively correlated with disease-free survival after surgery in hepatocellular carcinoma patients and SMAD3 expression in primary tumors (Fu et al. 2018). Regarding gastric cancer, recent studies have confirmed that small EVs derived from ascites of patients with gastric cancer can stimulate the mesothelial-to-mesenchymal transition, promoting peritoneal metastasis. Its Young’s modulus is negatively correlated with the invasiveness of gastric cancer cells and serve as a marker of the degree of malignancy in gastric cancer (Ge et al. 2024). EVs facilitate not only intracellular communication within the same organism but also interspecies interactions. The gut microbiota and exogenously ingested EVs, including those from plants and probiotics, play a significant role in the development of many diseases, including gastrointestinal and metabolic diseases (Díaz-Garrido et al. 2021; Niu et al. 2023; Molina-Tijeras et al. 2019). Recent research has found that EVs secreted by host cells and those ingested exogenously also affect the gut microbiota (Diaz-Garrido et al. 2021; Huang et al. 2023).

This review aims to delve into the intricate interactions between the host and the gut microbiota, particularly through the mediation of EVs, and how these interactions influence the development and progression of CRC. We will focus on analyzing the pivotal role of EVs in the information exchange between the host and gut microbiota, and examine the impact of this communication mechanism on CRC. Furthermore, this paper will also evaluate the potential of EVs from various sources as biomarkers and therapeutic targets, providing new insights into the pathobiology of CRC, thereby contributing to the scientific basis for future diagnostic and therapeutic strategies.

## Biological basis of EVs

### Classification, formation, and markers of EVs

At present, EVs refer to a class of membrane-enclosed vesicles secreted by cells. However, the scientific community still faces controversies regarding the classification and nomenclature of EVs (Pocsfalvi et al. 2016). There are currently two widely recognized naming conventions for EVs. The first, as suggested by the International Society for EVs, categorizes EVs based on their physicochemical properties such as size, density, and biochemical composition (Théry et al. 2018). The second classification is based on size, origin, formation process, and biological characteristics, traditionally dividing EVs into three main categories: exosomes, microvesicles, and apoptotic bodies (Table 1) (Shao et al. 2018; Bella 2022). Exosomes are vesicle-like bodies actively secreted by various living cells into the extracellular space. They originate from the endosomal system, undergoing stages including endocytic vesicles, early endosomes, intraluminal vesicles, and multivesicular bodies. Eventually, the multivesicular bodies fuse with the cytoplasmic membrane, releasing intraluminal vesicles into the extracellular space, thus forming exosomes (Kalluri and LeBleu 2020). Among the markers shown in Table 1, tetraspanins are small transmembrane proteins that mediate cell adhesion, migration, invasion, membrane fusion, and signal transduction (Andreu and Yáñez-Mó 2014). They are highly enriched in exosomes. Alix and TSG101 are part of the endosomal sorting complex required for transporting cargo into exosomes (Raiborg and Stenmark 2009). Microvesicles are formed by the budding of the cell membrane, hence their membrane composition is significantly similar to that of the parent cell (Shao et al. 2018). Some adhesion molecules, such as integrins, selectins, and CD40, are enriched in microvesicles (Shao et al. 2018; Bian et al. 2019). Apoptotic bodies, considered to be vesicular bodies formed during cellular shrinkage in apoptosis, are usually considered not attributed the function of transferring substances to recipient cells (Shao et al. 2018), unlike microvesicles and exosomes. Among its markers, phosphatidylserine is an anionic phospholipid located on the inner surface of normal cell or organelle membranes, but can flip to the outer surface during the early stages of apoptosis due to phospholipid scramblase activation (Calianese and Birge 2020). Additionally, during apoptosis, nuclear fragmentation occurs, and the fragments are encapsulated into apoptotic bodies. Thus, genomic DNA can serve as a marker of apoptotic bodies. It is worth noting that this traditional categorization has been updated as research has progressed. Newly discovered species of EVs, such as apoptotic vesicles, autophagic EVs, stressed EVs, and matrix vesicles (Sheta et al. 2023), have continued to appear on the scientists’ horizon. Given that these kinds of EVs were not mentioned in the studies covered in this review, they are not described in detail. However, in-depth studies of these categorized EVs may provide more insights into the intercellular communication of EVs and their role in CRC.

**Table 1:** Major types of EVs

vesicle	Formation process	size (nm)	density (g/mL)	origin	markers
exosomes	Formed by the fusion of multivesicular bodies with the cell membrane	40–200 (30–150)	1.13–1.18 (1.13–1.19)	endosomes	tetraspanins, Alix, TSG101
microvesicles	Formed by budding from the cell membrane	200–2000 (150–1000)	1.16–1.19	plasma membrane	integrins, selectins, CD40
apoptotic bodies	Formed by shrinking and fragmenting of cells during apoptosis	500–2000 (1000–5000)	1.16–1.28	plasma membrane, endoplasmic reticulum	phosphatidylserine, genomic DNA

Both gram-positive and gram-negative bacteria are known to secrete EVs, which, when originating from bacteria, are referred to as Bacterial EVs (BEVs) or Bacterial Membrane Vesicles (BMVs)(Ou et al. 2023; Toyofuku et al. 2019). The phenomenon of bacterial secretion of EVs was initially discovered through the bubbling of the outer membrane in gram-negative bacteria. Hence, BMVs secreted by gram-negative bacteria are often termed as Outer Membrane Vesicles (OMVs). Based on our knowledge, current studies on the impact of BMVs on CRC predominantly focus on OMVs, thus warranting a detailed discussion on OMVs. Since classical OMVs originate from the bubbling of the outer membrane, they are rich in outer membrane proteins (Orench-Rivera and Kuehn 2016). Additionally, their contents, comprising DNA, RNA, periplasmic and cytosolic proteins, and virulence factors, are enveloped by an outer leaflet of lipopolysaccharides and an inner leaflet of phospholipids from the gram-negative bacterial outer membrane (Toyofuku et al. 2019). It is noteworthy that other types of BMVs, including cell membrane vesicles produced by gram-positive bacteria, as well as outer inner membrane vesicles and tubular membrane structures, have been increasingly identified in recent years (Toyofuku et al. 2019). Research into the impact of BMVs on CRC requires further exploration.

### Mechanisms of EVs in intercellular communication

The EVs released by donor cells circulate in various body fluids, transporting to the recipient cells, thereby playing their role in intercellular communication. When EVs arrive near the recipient cells, they can perform their function of intercellular communication by signaling directly on the cell surface or by transferring their contents into the cell. Their contents include proteins, nucleic acids, lipids, and metabolites (Kalluri and LeBleu 2020). The process of cell-to-cell communication by EVs has specific cell-spectrum targeting. Most of this targeting acts through protein receptors and adhesion molecules on the surface of EVs. On the one hand, EVs can bind to and activate receptors and ligands expressed by the target cells through proteins on their surface and trigger intracellular signaling pathways without being taken up into the recipient cell (Mathieu et al. 2019). On the other hand, EVs can also transfer their contents into the recipient cell through direct fusion with the plasma membrane or active endocytosis by the recipient cells (French et al. 2017). Eventually, internalized EVs can release their contents and exert their effects under specific conditions through the indirect route of endosomal escape or fusion with the plasma membrane (Mathieu et al. 2019). Additionally, endosomes containing ingested EVs can bind to the cell nucleus or endoplasmic reticulum, transferring informational substances and thus playing a significant biological role (Santos et al. 2018; Corbeil et al. 2020). Apart from releasing their contents into the cytoplasm of the recipient cells for degradation, EVs can also complete their metabolic processes within the recipient cells by lysosomal degradation or direct release to the outside of the recipient cells (Liu and Wang 2023).

The effects of the intercellular communication function of EVs on cancer have been extensively studied. This review will continue with a description of EVs that change or play a role in CRC and its related processes during microbial-host interactions.

## Gut microbiota in the development of CRC

### The role of gut microbiota in CRC progression: focusing on intestinal barrier and immune system interactions

Gut microbiota is an important component of the tumor microenvironment, and several excellent reviews have described the relationship between gut microbiota and CRC (Wong and Yu 2019, 2023; Louis et al. 2014). Specifically, inflammation, immune regulation, metabolism of dietary components, and genotoxin production, all of which are CRC-associated pathogenic mechanisms, have been found to be closely related to the gut microbiota, and one of the most widely studied mechanisms is the gut microbiota that influences the development of CRC by interacting with the host’s intestinal barrier and immune system. This part will summarize the important literature in the related field in the last 5 years in order to illustrate how gut microbiota-dependent pathways bidirectionally regulate the host’s intestinal barrier and immune response.

On the one hand, in the case of imbalanced gut microbiota, host-gut microbiota interactions can disrupt the intestinal barrier and promote an inflammatory response. Myeloid cells in the colonic mucosa can recognize bacteria via Toll-like receptors in the presence of a compromised intestinal barrier, which in turn activates MyD88-dependent proinflammatory signaling pathways (Eftychi et al. 2019). Besides, commensal bacteria in mice inhibit the Hedgehog signaling pathway by activating Toll-like receptor-2 in epithelial cells, which in turn inhibits the Hedgehog signaling pathway and reduces nrp1 protein levels, leading to diminished intestinal barrier function (Pontarollo et al. 2023). In addition, *Fusobacterium nucleatum* is a bacterium significantly associated with CRC (Castellarin et al. 2012; Kostic et al. 2012), and its EVs can promote the loss of epithelial barrier by activating RIPK1 and RIPK3 and cause epithelial cell necrosis, leading to intestinal barrier disruption in colitis (Liu et al. 2021).

On the other hand, there are also some bacteria that can maintain the intestinal barrier and act as anti-inflammatory agents by interacting with the host. In this process, the cytokines IL-17 and IL-22 and the pathways related to their secretion have been shown to play an important role in several studies. Dendritic cells can recognize resident bacteria in the intestinal mucosa through the Mincle-Syk axis and stimulate intestinal T cells and innate lymphocytes to produce IL-17 and IL-22, which ultimately maintain and enhances the immune barrier function of the gut and prevents systemic inflammation (Martínez-López et al. 2019). IL-17 could also affect the composition of the gut microbiota, and thereby enhancing or restoring the integrity of the intestinal mucosal barrier (He et al. 2022). Furthermore, commensal bacteria, including epithelium-associated commensal segmented filamentous bacteria and *Escherichia coli* (GDAR2-2), can induce an increase in the number of type-3 innate lymphoid cells, which are capable of secreting IL-22, which in turn regulate IL-22-mediated natural immune responses and maintain intestinal barrier function (Talbot et al. 2020; Wu et al. 2022).

**Fig. 1:**
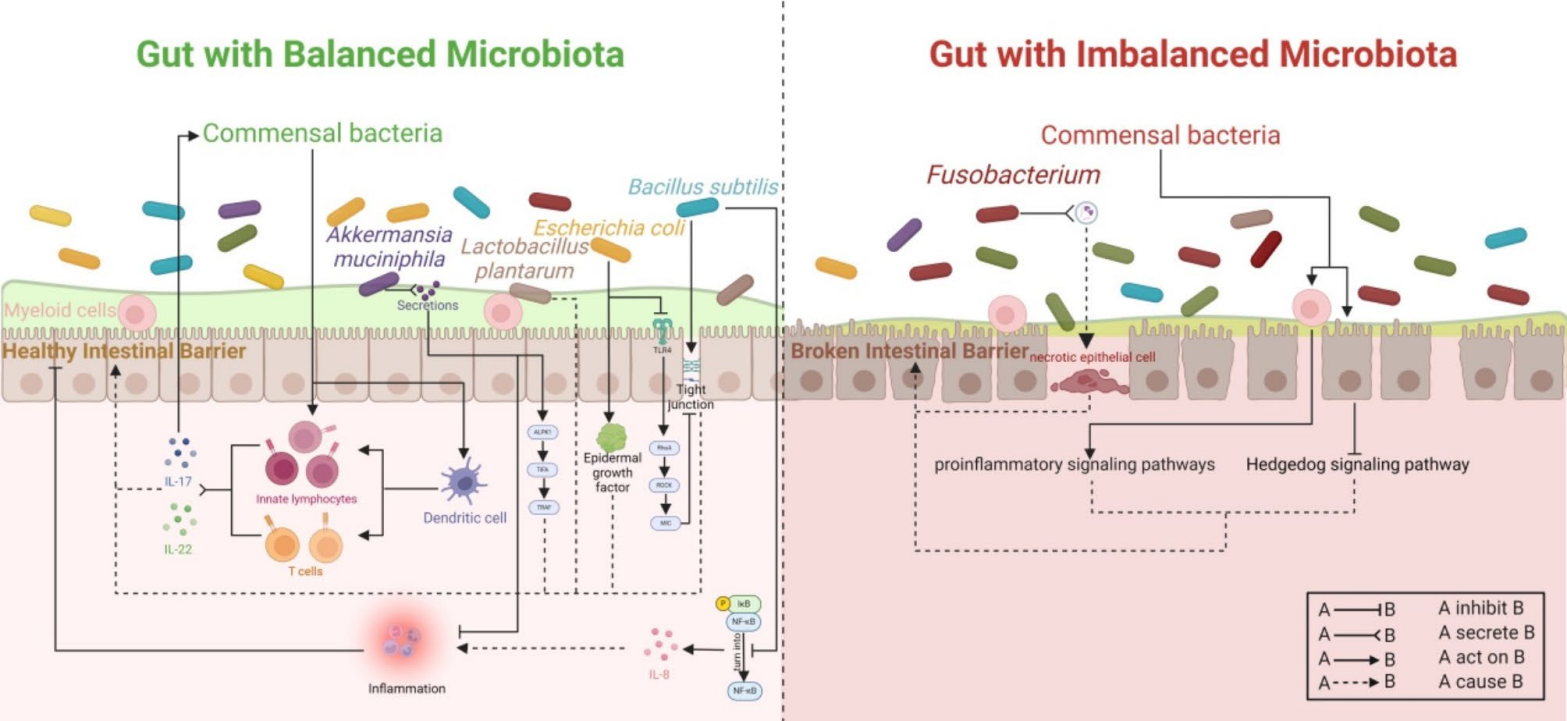
The interaction of gut microbiota with the intestinal barrier and immune system (Created with BioRender.com)

In addition, it is possible to modulate the gut microbiota by exogenously intaking probiotics, which in turn affects the intestinal barrier and the immune system. Several studies have been conducted to affirm the particular strains of *Bacillus subtilis*, *Lacticaseibacillus paracasei*, *Lactobacillus acidophilus*, *Lactobacillus casei*, *Lactobacillus plantarum*, and *Escherichia coli* (Nissle 1917) play a role as probiotics in maintaining or enhancing the intestinal barrier and limiting the inflammatory response (Rhayat et al. 2019; Wang et al. 2019; Yu et al. 2019; Xu et al. 2020, 2022; Li et al. 2021; Liu et al. 2022a, b; Chen et al. 2023; Xiao et al. 2024). Furthermore, *Akkermansia muciniphila*, a commensal bacterium inhabiting the intestinal mucosal layer, is also recognized for its probiotic potential. This bacterium contributes to maintaining the integrity of the intestinal barrier by releasing ADP-heptose related molecules, which activate the ALPK1/TIFA/TRAF6 pathway, enhancing the expression of genes vital for barrier function. Furthermore, it secretes β-acetylaminohexosidase (Amuc_2109), playing a key role in mitigating oxidative stress, excessive expression of inflammatory factors and inflammasomes, and reshaping the gut microbiota in colitis models, thereby preserving the barrier integrity and limiting intestinal inflammation (Martin-Gallausiaux et al. 2022; Qian et al. 2022). The interaction of gut microbiota on the intestinal barrier and immune system has been shown in Fig. 1.

The balanced gut microbiota can stimulate immune cells, ultimately promoting the release of IL-17 and IL-22 to maintain the intestinal barrier. IL-17, in turn, can regulate the composition of the gut microbiota. Additionally, various beneficial bacteria, either existing in the gut or acquired through external intake, also play a beneficial role in protecting the intestinal barrier and suppressing inflammation. An imbalanced gut microbiota is characterized by an increase in harmful bacteria and a decrease in beneficial bacteria. In this state, the gut microbiota can damage the intestinal barrier through various signaling pathways and induce an inflammatory environment.

### Dynamics of gut microbiota alterations throughout CRC development

The composition of the gut microbiota changes significantly during the development of CRC. Not only is there a significant difference in the gut microbiota between healthy individuals and CRC patients (Yang et al. 2019; Torres-Maravilla et al. 2021), but the composition of the gut microbiota of CRC patients with different TNM stages also varies (Liu et al. 2023a, b). Specifically, CRC patients showed a decrease in beneficial bacteria such as *Lactobacillus* and *Bifidobacterium* and an increase in harmful bacteria such as *Fusobacterium* and *Peptostreptococcus* compared to healthy individuals (Torres-Maravilla et al. 2021). For CRC with different TNM stages, it has been found that the diversity and evenness of gut microbiota in patients with stage III-IV CRC was significantly higher than that in patients with stage I-II CRC, and the genera *Proteus*, *Parabacteroides*, *Alistipes* and *Ruminococcus* were significantly enriched in the fecal microbiota of patients with stage III-IV CRC (Liu et al. 2023a, b). Differences in gut microbiota also exist between patients with left-sided and right-sided colon cancer. The abundances of *Streptococcus* and *Enterobacter* in the gut microbiota of left-sided colon cancer patients are higher than in right-sided colon cancer patients, and *Bifidobacterium* is significantly associated with left-sided colon cancer (Kneis et al. 2023). The gut microbiota in CRC patients is also influenced by age and race. Compared with CRC patients aged 65 years or younger, the abundance of *Bacteroides vulgatus* decreases in patients older than 65, while *Bacteroides fragilis* abundance increases (Wu et al. 2023). Although some studies have reported differences in the gut microbiota of CRC patients of different races (Elkholy et al. 2023), it is argued that causal inferences should be avoided in attributing these differences to specific racial or ethnic groups (Findley et al. 2016). Differences in microbiota among races are likely related to economic conditions, dietary patterns, etc., so this topic will not be discussed here. Furthermore, interventions aimed at restoring gut microbiota balance, including the intake of drugs and probiotics, have been shown to inhibit CRC progression and offer therapeutic benefits (Torres-Maravilla et al. 2021; Kvakova et al. 2022; Zhu et al. 2019; Gou et al. 2023). On the one hand, this suggests that the compositional characteristics of the gut microbiota could be used as a potential marker for colorectal carcinogenesis and for determining the degree of progression; on the other hand, this suggests that there is an interaction between the composition of the gut microbiota and the development of CRC, and that intervening the imbalanced gut microbiota may offer help in the treatment of CRC.

### Potential role of EVs in the diagnosis of CRC

Challenges and Advances in EVs Isolation, Purification, and Characterization.

As of now, there is no universally recognized optimal method for the isolation, purification, and characterization of EVs, which represents one of the significant challenges in EVs-related basic research and clinical applications. The primary reasons are the diversity of EVs, their varying degrees of overlap in size and physicochemical properties with other substances like lipoproteins, and the different advantages and disadvantages of various separation and characterization techniques.

Regarding common separation and enrichment methods for EVs, differential ultracentrifugation is the most extensively used technique. However, it is complex and time-consuming, making it unsuitable for clinical testing. This method, along with tangential-flow ultrafiltration and exclusion chromatography, faces challenges in completely separating EVs from other impurities (e.g., high-density lipoproteins, chylomicrons). The polymerization precipitation method is straightforward to operate, but maintaining the activity of EVs is challenging, which limits further analysis. The immunoaffinity capture method offers good specificity, but it enriches EVs based on their expression of specific surface proteins, the scope of its application has certain limitations (Stam et al. 2021; Yang et al. 2020a, b). Microfluidics, compared to other methods, offers numerous advantages, such as small sample volume requirements, fast analysis rates, and high separation purity. Its main drawback is the low sample capacity (Yang et al. 2020a, b; Havers et al. 2023). Overall, it presents a promising application prospect. Table 2 summarizes several commonly used separation and purification methods, as well as the advantages and disadvantages of each of these methods(Lobb et al. 2015; Zarovni et al. 2015; Brennan et al. 2020; Cheruvanky et al. 2007; Böing et al. 2014; Meggiolaro et al. 2022).

**Table 2:** Methods for the isolation and purification of EVs

Isolation and Purification Method	Advantages	Disadvantages
Differential Ultracentrifugation (84)	Widely used and considered the “gold standard,” with mature operational techniques	Complex operation, time-consuming, difficult to handle large sample volumes, and challenging to completely separate EVs from high-density lipoproteins.
Immunoaffinity Capture (85)	High specificity, capable of enriching specific types of EVs	Can only enrich EVs that express certain specific surface proteins each time.
Polymer Precipitation (86)	Simple to operate	The resulting EVs may contain impurities, and the activity of EVs is hard to guarantee.
Tangential Flow Ultrafiltration (87)	Simple operation, suitable for isolating EVs from large-volume samples like urine	Cannot differentiate between lipoproteins or chylomicrons of similar size to EVs.
Size Exclusion Chromatography (88)	Simple operation, unlikely to damage EVs, and can retain their activity	Similar to tangential flow ultrafiltration, and samples are diluted during the process.
Microfluidic Chip Separation (89)	Requires a small sample volume, fast separation speed, and high purity of separated EVs	High cost, technically demanding, and difficult to handle large sample volumes.

Techniques for characterizing EVs include microscopic imaging, dynamic light scattering techniques, flow cytometry, and methods for detecting their proteins and nucleic acids (Bella 2022). As far as we know, the biomarkers of CRC-associated EVs studied so far mostly relate to EVs with specific surface proteins and their transported proteins and nucleic acids. Therefore, this discussion focuses on introducing characterization techniques pertinent to the detection of these substances. Traditional protein detection methods include protein immunoblotting and enzyme-linked immunosorbent assay, while traditional nucleic acid detection methods mainly involve polymerase chain reaction amplification followed by detection through electrophoresis or real-time fluorescence measurement. These methods are characterized by large sample volume requirements, cumbersome operational techniques, and lengthy processes. In recent years, numerous new technologies have emerged, including microfluidics-based techniques, exosomal protein screening, and protein detection technologies for analyzing individual EVs (Liu et al. 2019; Yoshioka et al. 2014; Ko et al. 2021), as well as nucleic acid detection technologies based on microfluidics, novel sensors, and nano-flow cytometry (Liu et al. 2022a, b; Ramshani et al. 2019; Joshi et al. 2015), which have made EV detection faster, more sensitive, and more precise.

In summary, although there is no “gold standard” for the isolation, purification, and characterization of EVs, researchers can still choose the most suitable method for their EVs research by combining the characteristics of different technologies according to their specific goals. Furthermore, the ongoing search for more convenient, efficient, and cost-effective techniques for clinical applications, especially for the enrichment and detection of EVs, remains a key focus of future EVs research.

### EVs in blood as potential markers for CRC

A number of studies have been conducted to detect EVs and their carriers in blood as biomarkers for CRC. Both RNAs and proteins carried by EVs serve as biomarkers. For detecting microRNAs (miRNAs), serum exosomal levels of miR-150-5p, miR-139-3p, miR-874, miR-27b-3p, miR-193a and let-7 g can be used to diagnose the onset and extent of progression of CRC(Zou et al. 2019; Liu et al. 2020; Zhang et al. 2020; Dou et al. 2021; Cho et al. 2021). Gasparello et al. and Maminezhad et al. suggested that the diagnosis of CRC can be achieved by detecting multiple serum exosomal miRNAs (Gasparello et al. 2020; Maminezhad et al. 2020). In terms of circular RNAs, the detection of serum exosomal hsa-circ-0004771 levels in patients with CRC can be used to differentiate between healthy populations, patients with benign intestinal diseases, and patients with stage I/II CRC (Pan et al. 2019). Whereas has-circ-0004831 can predict the occurrence and prognosis of CRC (Xing et al. 2020), circ-133 can be used to monitor CRC tumor progression (Yang et al. 2020a, b), and hsa-circ-0004771 can be used to predict patients’ resistance to 5-FU (Qiao et al. 2023). In addition, Guo et al. demonstrated that simultaneous detection of long RNAs from multiple blood EVs sources can also differentiate between CRC, colorectal adenomas, and healthy individuals, as well as for colorectal adenomas screening and CRC prognosis prediction (Guo et al. 2022).

In addition to detecting RNAs, endothelial damage during adjuvant chemotherapy can be assessed in CRC patients by detecting proteins of EV origin in blood samples, such as angiostatin encapsulated by serum EVs (Bar-Sela et al. 2020). Proteins such as CD147 and TLN1 can be used for CRC screening (Moyano et al. 2021; Soloveva et al. 2023), while TIMP1 and ADAM17 can be used to predict CRC metastasis (Sun et al. 2021; Rao et al. 2022). Chang et al. proposed the simultaneous detection of multiple proteins for differentiating between normal individuals, as well as patients with early and advanced CRC (Chang et al. 2021).

In addition, the levels of specific EVs can be used as biomarkers for CRC. For example, blood levels of CD133^+^ EVs have been correlated with the presence of metastatic CRC, patient prognosis (overall survival), and objective response rate for first-line systemic therapies (Brocco et al. 2022); and CD147^+^ EVs have been used as a monitor of the response to therapy in patients with CRC (Moyano et al. 2021). Cancer stem cell-derived EVs complement cancer diagnosis and monitoring (Haldavnekar et al. 2022). Alternatively, exogenous aptamers have been used to screen for specific CRC-associated EVs, such as simultaneous detection of W3 target-positive circulating tumor cells and exosomes for CRC diagnosis and metastasis monitoring (Lu et al. 2023a, b).

However, it is worth noting that existing studies on monitoring blood-derived exosomes and carryover substances as biomarkers for CRC have mostly focused on substances of the host’s own origin and have rarely considered the detection of BEVs as biomarkers. Nevertheless, a recent study has begun to explore the potential of BEVs for CRC diagnosis and tumor load assessment (Ou et al. 2023). The study of BEVs and substances carried by them in human blood may serve as a valuable new direction for screening and identifying biomarkers for CRC.

### EVs in stool and urine as potential markers for CRC

Compared to the detection of EVs in blood, the release of tumor markers by luminal exfoliation into stool, which occurs earlier than vascular invasion (Northrop-Albrecht et al. 2022), and the noninvasive detection of stool and urine, have generated significant research interest in both assays as well. Although relevant studies are still in their early stages, two studies have shown that EVs of fecal origin may serve as markers for CRC diagnosis, staging, and determining prognosis (Park et al. 2021; Li et al. 2023a, b). In the area of urine testing, studies have also suggested that detection of urine-derived EVs or the proteins they carry may serve as biomarkers for CRC (Erozenci et al. 2019; Yoon et al. 2023). Meanwhile, one study proposed specific strategies: the study by Kim et al. proposed the diagnosis of CRC by simultaneous detection of metagenomic and metabolomic data of stool microbial EVs (Kim et al. 2020). Detailed information on CRC-related markers of EVs origin is summarized in Table 3.

**Table 3:** Potential biomarkers for CRC in EVs

Authors	Category of Biomarkers	Name of Biomarkers	Source of EVs	Methods for the isolation	Description of EVs	Potential Function
Zou et al. (96)	miRNA	miR-150-5p	serum	polymer precipitation	exosomes	A potential biomarker for CRC diagnosis, differentiation, lymph node metastasis, TNM staging, and prognosis
Liu et al. (97)	miRNA	miR-139-3p	plasma	polymer precipitation	exosomes	A potential biomarker for early diagnosis and monitoring of metastasis in CRC
Zhang et al. (98)	miRNA	miR-874	serum	polymer precipitation	exosomes	A potential biomarker to distinguish CRC patients from healthy controls, as well as from patients with benign adenomas, and to be associated with distant metastasis, lymph node metastasis, differentiation, and TNM staging of CRC
Dou et al. (99)	miRNA	miR-27b-3p	blood	differential ultracentrifugation	epithelial-mesenchymal transition-cancer cells-derived exosomes	Overexpressed in CRC tissues and may serve as a biomarker for CRC metastasis
Cho et al. (100)	miRNA	miR-193a and let-7 g	plasma	polymer precipitation	exosomes	Potential biomarkers for the diagnosis, progression, and prognosis of CRC
Gasparello et al. (101)	miRNA	Combining nine miRNAs^a^	blood	differential ultracentrifugation	exosomes	For early diagnosis of CRC
Maminezhad et al. (102)	miRNA	Combining six miRNAs^b^	serum	differential ultracentrifugation	exosomes	For the diagnosis and staging of CRC
Pan et al. (103)	circ-RNA	hsa-circ-0004771	serum	differential ultracentrifugation	exosomes	Differentiate among healthy individuals, patients with benign intestinal diseases, and patients with stage I/II CRC
Xing et al. (104)	circ-RNA	has-circ-0004831	blood	EVs data are from the exoRBase database	exosomes	Upregulated in the blood exosomes of CRC patients and may predict the prognosis of CRC
Yang et al. (105)	circ-RNA	circ-133	plasma	differential ultracentrifugation	exosomes	Enriched in plasma exosomes of CRC patients, as a potential biomarker to monitor CRC progression and may serve as a therapeutic target
Qiao et al. (106)	circ-RNA	hsa-circ-0004771	serum	differential ultracentrifugation	exosomes	A potential predictive biomarker for 5-FU resistance in CRC patients
Guo et al. (107)	long RNAs	Combining seventeen long RNAs^c^	blood	differential ultracentrifugation	EVs	Differentiate CRC, colorectal adenoma, and healthy individuals, and used for colorectal adenoma screening and prognosis prediction in CRC
Bar-Sela et al. (108)	protein	Angiostatin	blood	differential ultracentrifugation	EVs	Evaluate endothelial damage during adjuvant chemotherapy in CRC patients
Moyano et al. (109)	protein and EVs	CD147 and CD147^+^ EVs	plasma	differential ultracentrifugation	CD147^+^ EVs	CD147 expressed in circulating EVs: CRC screening; CD147^+^ EVs: monitoring the response of CRC patients to treatment
Soloveva et al. (110)	protein	TLN1, ITGB3, TUBA4A, HSPG2	plasma	polymer precipitation	EVs	Potential biomarkers for the diagnosis of CRC
Sun et al. (111)	protein	ADAM17	serum	differential ultracentrifugation	exosomes	A potential biomarker for colorectal cancer metastasis
Rao et al. (112)	protein	TIMP1	serum	differential ultracentrifugation	EVs	Circulating biomarkers for non-invasive preoperative risk stratification in colorectal liver metastasis patients
Chang et al. (113)	protein	Combining six proteins^d^	serum	size exclusion chromatography	small EVs	Differentiate between normal controls, early-stage, and late-stage CRC patients
Brocco et al. (114)	EVs	CD133^+^ EVs	blood	no separate isolation step for EVs	EVs	Related to the presence of metastatic CRC, patient prognosis (Overall survival), and objective response rate for first-line systemic therapies
Haldavnekar et al. (115)	EVs	Cancer Stem Cell Derived EVs	plasma	differential ultracentrifugation	EVs	As a complementary tool for large-scale validation of clinical samples for existing cancer diagnosis, therapy monitoring, and longitudinal disease surveillance
Lu et al. (116)	circulating tumor cells and exosomes	W3 target-positive circulating tumor cells and exosomes	blood	differential ultracentrifugation	exosomes	Detecting W3-target-positive circulating tumor cells and exosomes for diagnosis and metastasis monitoring in CRC
Ou et al. (42)	EVs	BEVs	blood	differential ultracentrifugation	BEVs	Potential biomarkers for diagnosis of CRC and tumor burden assessment
Northrop-Albrecht et al. (117)	EVs	stool-derived EVs	stool	differential ultracentrifugation and polymer precipitation	EVs	Using Size Exclusion Chromatography to detect fecal EVs may as a preferred separation method for potential new biomarkers for early CRC detection
Park et al. (118)	EVs	microbe-derived EVs	stool	filtration and centrifugation	EVs	Potential biomarkers for detection, staging of CRC, and prediction of CRC prognosis
Li et al. (119)	EVs	stool-derived bacterial EVs	stool	differential ultracentrifugation and size exclusion chromatography	EVs	Potential biomarkers for personalized medicine and research
Erozenci et al. (120)	protein	Not exactly	urine	capture EVs by using VN-96 peptide	exosomes	Proteins from urine-derived EVs may serve as biomarkers for cancers such as CRC
Yoon et al. (121)	EVs	BEVs	urine	filtration and centrifugation	BEVs	Potential biomarkers for the diagnosis of CRC
Kim et al. (122)	metabolites and bacterial genera	Combining two metabolites and two bacterial genera^e^	stool	filtration and centrifugation	EVs	For the diagnosis of CRC

a Contains miR-144-5p, miR-144-3p, miR-486-5p, miR-15b-5p, miR-221-3p, miR-425-3p, miR-584-5p, miR-10a-5p, miR-483-5p, miR-141-3p, miR-222-3p, and miR-1247-5p

b Contains miR-19a, miR-20a, miR-143, miR-145, miR-150, and let-7a

c Contains HIST2H2AA4, H2BFS, UQCRHL, XCL2, AC008269.1, DMC1, RAB6D, KLHDC8B, CA3, APOL4, HIST1H2AI, ANKAR, SGMS1, CYP20A1, HIST1H2BB, STK3, and CBWD1

d Contains GCLM, KEL, APOF, CFB, PDE5A, and ATIC

e Two metabolites: leucine and oxalic acid; two bacterial genera: Collinsella and Solanum melongena

In summary, although it has been shown that BEVs in blood, stool, and urine can be used as potential biomarkers for CRC (Ou et al. 2023; Park et al. 2021; Li et al. 2023a, b; Yoon et al. 2023; Kim et al. 2020), research in this area is still in its infancy, and there are still many areas to be explored. Currently, there are still very few studies focused on detecting EVs extracted from urine. Additionally, current detection strategies do not specify which nucleic acids or proteins need to be detected, and the detection cost remains high, making clinical application a distant goal. Fecal EVs are closely related to those derived from gut microbiota. Fecal detection is non-invasive, which appears to be an ideal medium for detecting biomarkers. However, significant differences exist between the types and abundances of microorganisms identified in fecal samples and those found in the colon tissue of patients (Gamage et al. 2024). Therefore, relying solely on stool-derived exosomes may miss important microbial markers more closely related to tumor development and progression. In comparison, although detecting EVs in blood may cause more discomfort than using urine or stool, it is still more acceptable to patients than tissue extraction. Furthermore, blood research is the most comprehensive, and blood can also detect other important markers related to CRC progression beyond EVs. Therefore, blood detection may offer better application prospects. As shown in Table 3, the detection of EVs in blood predominantly uses the differential ultracentrifugation method. Additionally, due to their simplicity, Polymer Precipitation and Size Exclusion Chromatography are also commonly used by research institutions. For large-volume samples like urine and stool, separating and purifying EVs is more challenging. Researchers typically employ preliminary separation of EVs, combinations of multiple methods, or novel separation techniques. Further exploration is needed in basic research on detecting EVs in patient body fluids and in clinical translation.

## The influence of gut microbiota on CRC via EVs

### The role of gut microbiota-derived EVs in CRC

EVs of gut microbiota origin can influence CRC development in a variety of ways. Enterotoxigenic *Bacteroides fragilis* can secrete particles that stimulate the intestinal epithelium to produce exosome-like nanoparticles derived from the intestinal mucosa, influencing the recruitment and proliferation of T-helper type 17 cells in the intestine to promote colon cancer. Additionally, it influences the cell-secreted exosomal miR-149-3p to promote the differentiation of T-helper type 17 cells, thereby promoting CRC (Deng et al. 2015; Cao et al. 2021). The OMVs of *Fusobacterium nucleatum* may promote CRC by creating a pro-inflammatory environment, promoting mitochondrial fusion and cell invasion, and altering the expression levels of proteins from OMVs in an acidic tumor microenvironment (Lamprinaki et al. 2021; Lin et al. 2021; Zhang et al. 2023a, b). Other bacteria, such as Firmicutes and Proteobacteria, may also contribute to amino acid accumulation and energy depletion, reflected by EVs secreted by gut microbiota, through the altered composition in CRC (Kim et al. 2020). Furthermore, Vdovikova et al. showed that membrane vesicles from pathogenic and commensal bacteria have a global impact on the gene expression of colon carcinoma cells, thereby promoting the development of colon cancer (Vdovikova et al. 2018).

However, EVs secreted by gut microbiota do not necessarily promote the development of CRC and may also play a positive role in its treatment. For example, Aly et al. showed that the OMVs of *Salmonella Typhimurium* ATCC 14028 were verified to have potential antitumor activity in vitro (Aly et al. 2021). Kim et al. demonstrated that OMVs derived from genetically modified *Escherichia coli* (*Escherichia coli msbB*^*−/−*^), modified to avoid potential adverse effects due to bacterial endotoxin lipopolysaccharide) can suppress established tumors and prevent tumor metastasis through an interferon-γ mediated antitumor response, validated in the murine colon adenocarcinoma cell lines (Kim et al. 2017). Additionally, Vdovikova et al. showed that a small number of genes (e.g., RND3, TACSTD2, SOCS2, and ARL5B) are differentially regulated by membrane vesicles of pathogenic and non-pathogenic bacteria in the gut(Vdovikova et al. 2018), and SOCS2 has been found to be possibly associated with the tumorigenic potential of CRC (Kim et al. 2018), suggesting that some bacteria in the gut may inhibit CRC development. These studies demonstrate that EVs released by some bacteria may have a positive effect on CRC treatment, but their antitumor effects need further experimental verification. A summary of the studies on EVs of intestinal microbial origin affecting CRC is presented in Table 4.

**Table 4:** Potential role of gut microbiota-derived EVs in CRC Development

Authors	Specific EVs Studied	Source Bacteria of EVs	Overall impact on the development of CRC	Potential Role of EVs in CRC Development
Deng et al. (124)	enteropathogenic bacteria-secreted particles	enterotoxigenic *Bacteroides fragilis*	Promote the development of CRC	Enteropathogenic bacteria-secreted particles derived from *Bacteroides fragilis* stimulate intestinal epithelium to produce intestinal mucosa-derived exosome-like nanoparticles, promoting the recruitment and proliferation of T-helper type 17 cells, thereby facilitating colon cancer development
Cao et al. (125)	exosomes derived from Enterotoxigenic *Bacteroides fragilis*-inoculated cells	enterotoxigenic *Bacteroides fragilis*	Promote the development of CRC	Exosomal miR-149-3p from Enterotoxigenic *Bacteroides fragilis*-treated cells (HCT116 and SW480) enhances T-helper type 17 cell differentiation. This bacterium’s influence on colorectal carcinogenesis involves down-regulating miR-149-3p and promoting PHF5A-mediated RNA alternative splicing of KAT2A in CRC cells
Lamprinaki et al. (126)	*Fusobacterium nucleatum*-derived OMVs	*Fusobacterium nucleatum*	Promote the development of CRC	*Fusobacterium nucleatum*-derived OMVs play a role in CRC progression by creating a pro-inflammatory environment, thereby influencing the disease course
Lin et al. (127)	EVs released from *Fusobacterium nucleatum*	*Fusobacterium nucleatum*	Promote the development of CRC	EVs released from *Fusobacterium nucleatum* promote mitochondrial fusion and cell invasion in CRC cells, contributing to the disease’s progression
Zhang et al. (128)	OMVs from *Fusobacterium nucleatum*	*Fusobacterium nucleatum*	Promote the development of CRC	OMVs produced by *Fusobacterium nucleatum* experience significant changes in protein expression in the acidic tumor microenvironment, impacting the development and progression of CRC
Vdovikova et al. (129)	bacterial membrane vesicles from pathogenic bacteria *Vibrio cholerae* and non-pathogenic commensal bacteria *Escherichia coli*	pathogenic bacteria *Vibrio cholerae* and non-pathogenic commensal bacteria *Escherichia coli*	Promote the development of CRC. Some genes with altered expression might play a role in inhibiting CRC progression.	Membrane vesicles from both pathogenic bacteria *Vibrio cholerae* and non-pathogenic commensal bacteria *Escherichia coli* have a significant global impact on the gene expression of colon carcinoma cells
Aly et al. (130)	the OMVs of *Salmonella Typhimurium* ATCC 14,028	*Salmonella Typhimurium* ATCC 14,028	Inhibit the development of CRC	The OMVs of *Salmonella Typhimurium* ATCC 14,028 exhibit potential antitumor activity, suggesting their use as a promising safe antitumor immunotherapy or as an adjuvant to conventional chemotherapeutic drugs
Kim et al. (131)	OMVs derived from genetically modified *Escherichia coli*	genetically modified *Escherichia coli* (*E. coli msbB-/-*)	Inhibit the development of CRC	OMVs from genetically modified *Escherichia coli* (*E. coli msbB-/-*) show potential in suppressing established tumors and preventing tumor metastasis through an interferon-γ mediated antitumor response
Kim et al. (122)	microbial EVs	*Firmicutes* and *Proteobacteria*	Inhibit the development of CRC	The altered composition of *Firmicutes* and *Proteobacteria* in CRC contributes to amino acid accumulation and energy depletion, which is reflected in the properties of EVs secreted by these gut microbiota

In addition, exogenous intake of probiotics by the host may also alter the composition of the gut microbiota and inhibit the development of CRC through their secreted EVs. We would like to explain this topic in the section “The Mediating Role of EVs in Probiotic Influence on CRC”.

### Gut microbiota influences CRC development by affecting host cell-derived EVs

Gut microbiota can bidirectionally regulate CRC development by influencing the secretion and composition of host cell-derived EVs. A subset of studies suggests that this pathway can promote CRC development; Gu et al. found that intestinal dysbacteriosis promotes tumor exosome secretion in CRC xenograft mice, which in turn promotes the growth of xenograft tumors in vivo, leading to shortened survival time. The use of exosome secretion inhibitors can partially restore these adverse effects (Gu et al. 2020). Chang et al. showed that *Parvimonas micra* could promote CRC cell proliferation and survival by up-regulating the expression of miR-218-5p in cells and exosomes, thereby inhibiting the expression of its target gene, PTPRR, ultimately activating the Ras/ERK/c-Fos signaling pathway, and correlating with a poor prognosis in patients (Chang et al. 2023).

Other studies have shown that this modality can inhibit CRC development and may serve as a potential therapeutic modality. Jiang et al. found that lipopolysaccharide could alter the miRNA expression profile in exosomes of the human CRC cell line HCT-116, inhibit CRC cell invasion and migration via miR-200c-3p in exosomes, and promote apoptosis (Jiang et al. 2020). Commonly used in studies of intestinal absorption and cancer, Caco-2 cells are a human colon cancer cell line. Cytolethal distending toxin, a virulence factor from gram-negative pathogenic bacteria, targets the endo-lysosomal compartment in Caco-2 cells and disseminates through EVs secreted by these cells. This process leads to cell cycle arrest and cell death in cancer cells, suggesting the potential of these EVs in CRC therapy (Montanari et al. 2022).

## Host effects on CRC via EVs secreted by cells and substances in vivo or exogenously ingested

### The influence of the host on gut microbiota, gut, and CRC via EV-mediated pathways

EVs produced by the host’s own cells can influence the gut microbiota. A review has elaborated on miRNAs, small molecules that play an important role in the process of cellular communication, which are largely dependent on the loading of EVs (Sarshar et al. 2020). The focus here is on the role played by miRNAs that have been experimentally shown to be loaded by EVs. Several studies have identified significant roles for exosomes from MSCs, which have been found to exert anti-inflammatory effects, modulate gut microbiota, promote intestinal barrier function, and potentially reverse cancer in mice with colitis (Gu et al. 2021; Yan et al. 2023; Ocansey et al. 2022), with some of these roles possibly related to exosome-loaded miR-181a (Gu et al. 2021). MiR-200b-3p derived from colonic EVs can restore the intestinal barrier and alleviate colitis, not only by directly affecting the number of specific bacteria in the gut microbiota but also by altering the abundance of different genera in the microbiota, thus changing the composition of BMVs secreted by the microbiota (Shen et al. 2022). Moreover, adherent-invasive *Escherichia coli* (AIEC LF82), a bacterium that adheres to and invades intestinal epithelial cells, inducing a pro-inflammatory response, can, after infecting the organism, induce intestinal epithelial cells to secrete miR-30c- and miR-130a-containing exosomes. These exosomes inhibit the autophagic response, thereby reducing the host’s intracellular clearance of the bacterium (Larabi et al. 2020).

Similarly to how bacterial-derived EVs can influence host intestinal health and CRC development, the host can secrete substances that counteract the secretion of EVs by the gut microbiota. Sodium taurocholate, one of the bile acids secreted by the liver, can increase the production and composition of Campylobacter jejuni-derived OMVs (Davies et al. 2019). Sialic acid-binding Ig-like lectin 7, a protein predominantly secreted by immune cells, was found to interact with OMVs from *Fusobacterium nucleatum*, leading to a pro-inflammatory phenotype in human monocyte-derived dendritic cells and inducing a tumor-associated phenotype in human monocyte-derived macrophages, contributing to the formation of an inflammatory environment, an important factor in CRC development (Lamprinaki et al. 2021). However, although it has been demonstrated that normal human cells can influence bacterial EVs by affecting the composition of gut microbiota and CRC cells can also influence gut microbiota-derived EVs, no direct evidence has been found that normal human cells can influence CRC by directly affecting the EVs of a specific gut microbe through substance secretion. Further studies are required to confirm whether this pathway influences CRC development.

### The mediating role of EVs in probiotic influence on CRC

EVs play an important role not only in the relationship between endogenous gut microbiota and CRC, but also in the process by which the effects of hosts’ ingestion of exogenous probiotic bacteria, especially those from the *Lactobacillaceae* family, affect CRC. *Lactobacillus plantarum*-derived EVs can restore the chemosensitivity of HCT116 cells and 5-fluorouracil-resistant CRC cells through the PDK2 signaling pathway (An and Ha 2022). *Lactobacillus rhamnosus* GG-derived EVs can modulate intestinal immunity and gut microbiota, and enhance the efficacy of anti-PD-1 immunotherapy for CRC in mice (Lu et al. 2023a, b). *Lacticaseibacillus paracasei*-derived EVs can inhibit growth and induce apoptosis of CRC cells through the PDK1/AKT/Bcl-2 signaling pathway both in vitro and in vivo (Shi et al. 2021).

In addition to *Lactobacillaceae*, EVs derived from *Akkermansia muciniphila* can maintain the integrity of the intestinal barrier, restore gut microbiota dysbiosis, and inhibit the development of CRC by enhancing anti-programmed cell death protein 1 therapy, inducing the secretion of HSP70, and promoting a Cytotoxic T Lymphocytes-associated immune response through the acetyltransferase it carries (Jiang et al. 2023; Wang et al. 2023).

Additionally, the potential to inhibit CRC could be further enhanced by engineering EVs. Liu et al. increased the production of membrane vesicles in *Escherichia coli* (MG1655) by establishing an “autonomous controlled peptidoglycan hydrolase expression system”, resulting in a higher activation of the innate immune response in HCT116 (Liu et al. 2023a, b). Furthermore, probiotics can also affect CRC cell-derived EVs. *Kefiri SGL 13*, a probiotic shown to inhibit CRC, may modify the protein composition of EVs secreted by HT-29 cells following co-culture, thereby promoting apoptosis and anti-inflammatory effects (Brandi et al. 2019).

### The impact of milk-derived and plant-derived EVs on CRC

In addition to probiotics, the intake of milk-derived and plant-derived EVs can have a positive effect on the composition of the gut microbiota and on CRC as well. Milk-derived EVs can alter the composition of the gut microbiota, elevating the beneficial bacteria such as *Lachnospiraceae* family, *Akkermansia*, and others, which are known to have an inhibitory effect on the development of CRC (Jiang et al. 2023; Wang et al. 2023; Zhang et al. 2023a, b). They also reduce the primary tumor burden in CRC-implanted mice (Samuel et al. 2021). Additionally, these EVs decrease the number of harmful bacteria, such as *Verrucomicrobiaceae* family and *Desulfovibrio* (Du et al. 2021; Zhou et al. 2019). Moreover, they are important in maintaining the integrity of the intestinal mucosa, improving the intestinal barrier function, and modulating intestinal immunity (Du et al. 2021, 2022; Tong et al. 2020; Maghraby et al. 2021). However, although not observed in CRC mice, animal experiments have shown that milk-derived EVs promote pancreatic and breast cancer metastasis by inducing epithelial-mesenchymal transformation of cancer cells in the presence of primary tumors. This metastasis-promoting effect was reversed by the oral administration of milk-derived EVs after the removal of the primary tumor(Samuel et al. 2021). Therefore, the safety, efficacy, and timing of milk-derived EVs for CRC treatment need further confirmation.

The study of plant-derived EVs affecting host health through the gut microbiota has also received much attention in recent years. Tomato-derived EVs can promote the growth of probiotic *Lactobacillus* species while inhibiting the growth of *Clostridioides difficile*, *Fusobacterium nucleatum*, and other harmful bacteria(Lee et al. 2023). These bacteria have been shown to be associated with CRC development (Castellarin et al. 2012; Lamprinaki et al. 2021; An and Ha 2022; Lu et al. 2023a, b; Brandi et al. 2019; Anderson and Sears 2023). Zu et al. extracted exosome-like nanotherapeutics from tea and found that they significantly increased the community abundance and species diversity of the gut microbiota of mice, as well as maintained the colon barrier and inhibited the development of colon tumors (Zu et al. 2021). *Paris polyphylla*, a traditional Chinese medicine, has not been shown to influence CRC development with its secreted EVs, but one study found that its active ingredient reversed the promotion of mitochondrial fusion and migration in CRC cells by *Fusobacterium nucleatum*-derived EVs (Lin et al. 2021). This suggests that active components derived from plants may inhibit the promotion process of CRC by EVs of intestinal harmful bacterial origin, and such studies need to be further explored.

## Prospects and challenges of EVs in CRC applications

### Potential applications of EVs in CRC prevention, diagnosis, and treatment

**Fig. 2:**
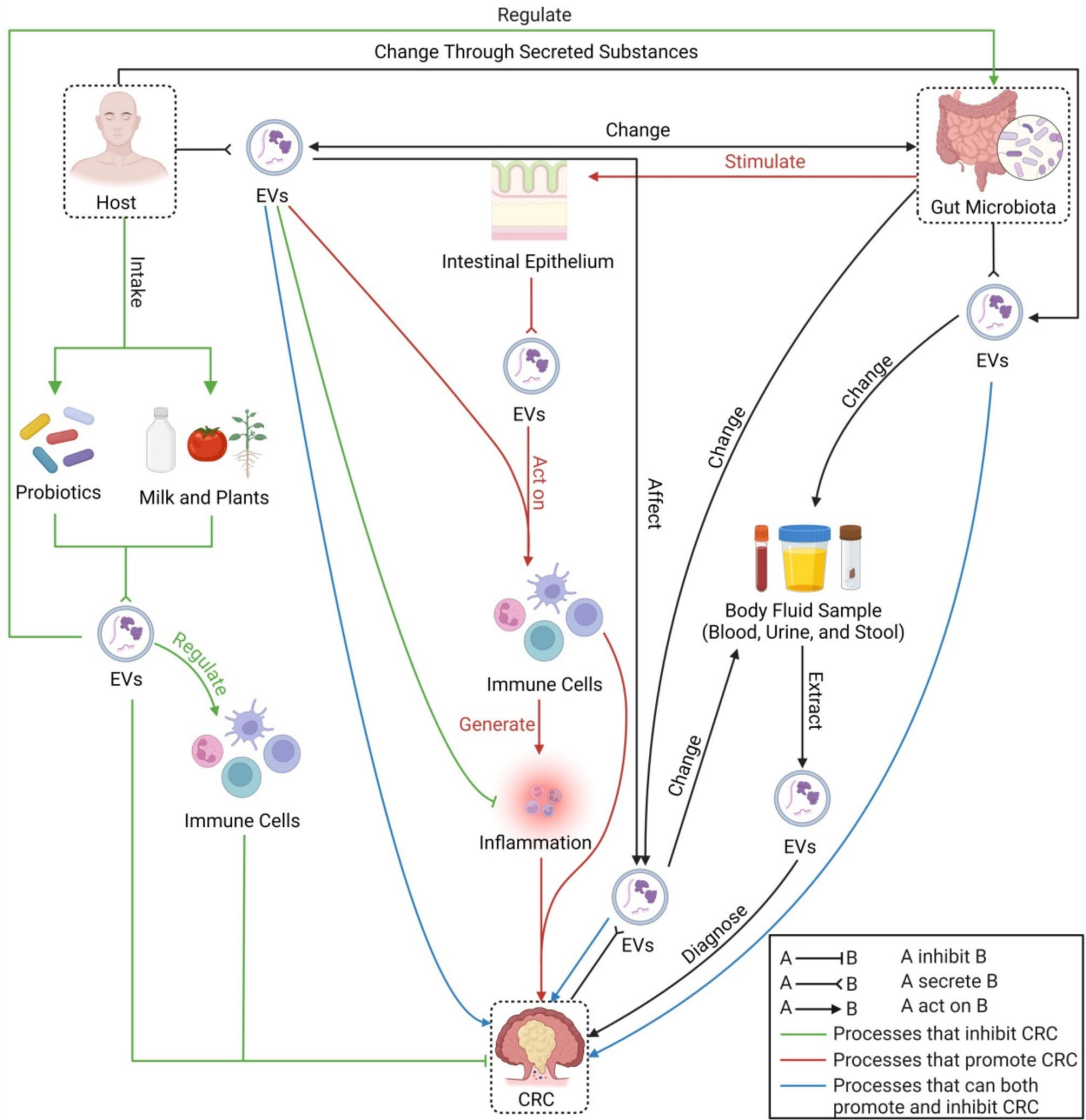
Pathways that influence CRC via host-gut microbiota interactions mediated by EVs (Created with BioRender.com)

The burgeoning research on EVs has revealed their potential as biomarkers and therapeutic targets for CRC. Pathways through which host-gut microbiota influence CRC via interactions mediated by EVs have been integrated into Fig. 2. The potential applications of these EVs in the clinical management of colorectal cancer will be summarized in terms of prevention, diagnosis, and treatment.

This figure illustrates the complex host-gut microbiota interactions mediated through EVs and how these interactions relate to CRC. On the one hand, exogenous substances ingested by the host and a healthy gut microbiota can inhibit the development of CRC through various EVs-mediated pathways; on the other hand, an imbalanced gut microbiota can promote the development of CRC through other EVs-mediated pathways. In addition, CRC can be diagnosed by detecting EVs in different body fluids.

EVs that mediate host-gut microbiota crosstalk can be used as a strategy to prevent CRC. By using liquid biopsies to detect changes in gut microbiota through microbiota-derived EVs, it is possible to predict CRC risk and enable early intervention(Zhang et al. 2020; Gasparello et al. 2020; Pan et al. 2019; Guo et al. 2022). Moreover, consuming foods enriched with beneficial EVs can prevent CRC by positively influencing the gut microbiota’s composition and function(Lee et al. 2023; Zu et al. 2021), offering simplicity and ease of acceptance.

EVs that mediate host-gut microbiota crosstalk can be used as biomarkers for CRC screening and diagnosis. EVs with specific surface proteins and EV-associated biomolecules (including nucleic acids and proteins), can be used as CRC-associated biomarkers, providing a non-invasive means of detection. These biomarkers have the potential not only to differentiate healthy individuals from those with CRC but also to identify early-stage CRC and distinguish it from other benign gastrointestinal disorders(Ou et al. 2023; Zhang et al. 2020; Pan et al. 2019; Guo et al. 2022; Soloveva et al. 2023; Erozenci et al. 2019; Yoon et al. 2023; Kim et al. 2020). Furthermore, EVs can provide a basis for staging CRC, monitoring CRC metastasis and drug treatment effects, and predicting prognosis(Liu et al. 2020; Zhang et al. 2020; Dou et al. 2021; Cho et al. 2021; Maminezhad et al. 2020; Xing et al. 2020; Yang et al. 2020a, b; Guo et al. 2022; Sun et al. 2021; Chang et al. 2021; Haldavnekar et al. 2022).

EVs that mediate host-gut microbiota crosstalk have potential for the treatment of CRC. If therapeutically significant EVs can be produced and isolated cost-effectively and on a large scale, they may offer more precise treatments with fewer side effects for CRC. Additionally, enhancing the production of specific EVs by modifying them or the cells that produce them to enhance their targeting properties(Xiong et al. 2023), or packaging other drugs within EVs to provide a synergistic anti-tumor effect, is feasible. Finally, incorporating plants, milk, and probiotics that secrete therapeutically useful EVs into a patient’s daily diet provide a simplified, accessible, and long-term adjuvant therapy(An and Ha 2022; Lu et al. 2023a, b; Shi et al. 2021; Jiang et al. 2023; Wang et al. 2023; Liu et al. 2023a, b; Brandi et al. 2019; Zhang et al. 2023a, b; Samuel et al. 2021; Du et al. 2021, 2022; Zhou et al. 2019; Tong et al. 2020; Maghraby et al. 2021; Lee et al. 2023; Zu et al. 2021). It should be especially noted by researchers that even for the same species of bacteria, different strains may have completely opposite effects on the body. For example, some strains of *Escherichia coli* can keep the normal intestinal barrier, while other strains can damage the intestinal barrier, thereby triggering inflammation and promoting the occurrence and development of CRC (Pleguezuelos-Manzano et al. 2020; Viswalingam et al. 2020). Therefore, when selecting probiotics for research, special attention should be paid to the differences in the effects of different strains on the body. In addition, blocking the pathways associated with EVs that promote CRC development may also be beneficial in treating CRC.

### Challenges in EVs for CRC

**Fig. 3:**
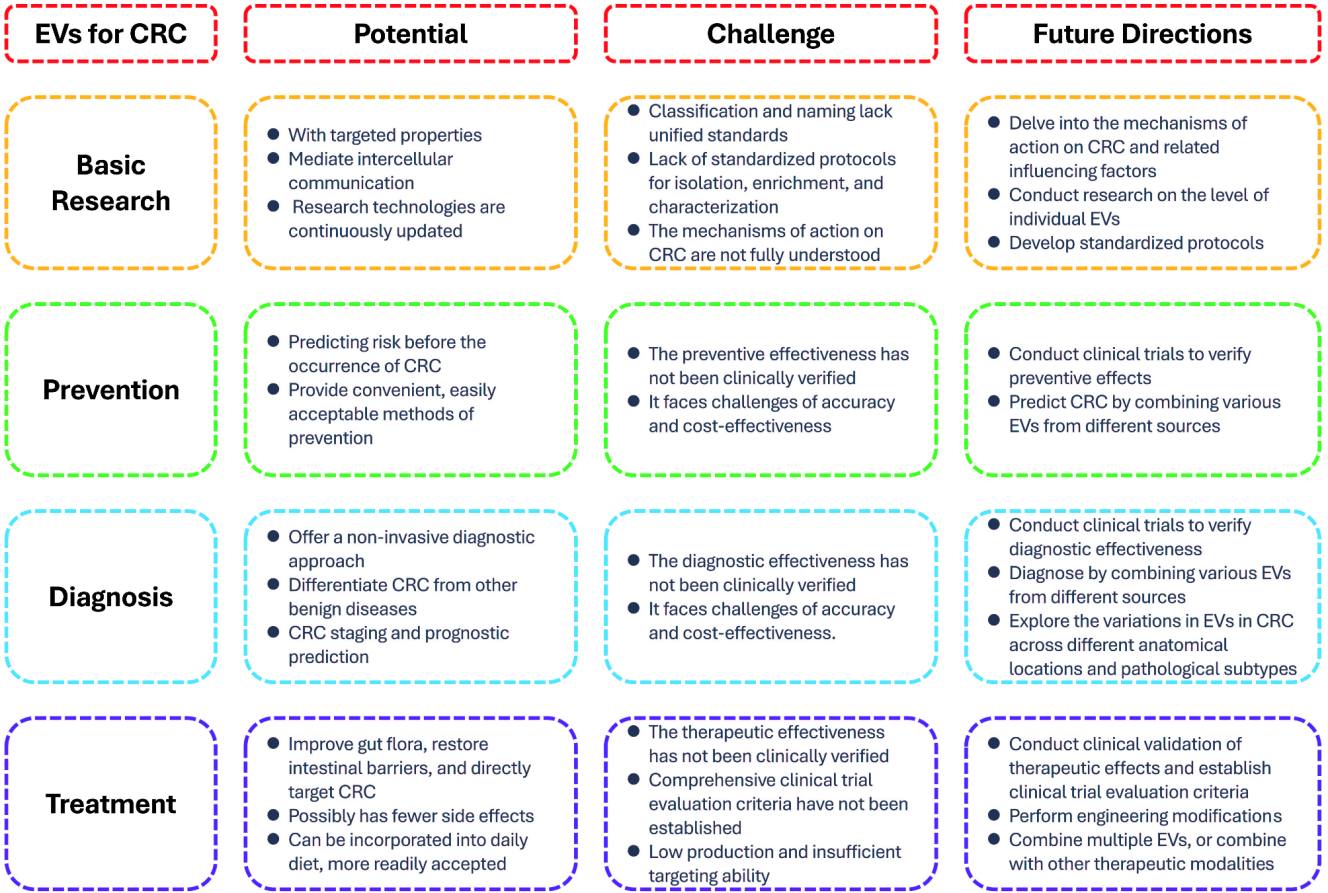
The summary of prospects and challenges of EVs in CRC applications

Although current research suggests that EVs have potential for use in CRC-related clinical procedures, a considerable gap remains before their practical application in the clinic. Firstly, there is a lack of recognized standard methods for the isolation, enrichment, and characterization of EVs. Therefore, establishing universally accepted protocols is crucial for clinical translation. Secondly, due to the diversity of EVs, identifying, characterizing, and isolating the most valuable EVs for CRC-related clinical applications from the numerous EVs secreted by various cell types remains a difficult task. Furthermore, some studies have only demonstrated that specific EVs can affect the development of CRC and related pathological processes, but more in-depth mechanistic studies are lacking. Moreover, most studies are limited to animal and cellular experiments, lacking clinical trial data on the effectiveness and safety of EVs involved in host-microbiota interactions when applied to CRC patients. Finally, natural EVs may exhibit insufficient targeting and low yield, posing challenges to their efficacy and cost-effectiveness in CRC therapy. Some specific research directions to address these challenges are shown in Fig. 3.

This Figure provides a concise and intuitive summary of the potential, challenges, and future research directions of EVs for CRC-related basic research, prevention, diagnosis, and treatment.

## Conclusion

EVs, which carry a wide range of macromolecules and possess certain targeting properties, are important mediators of host-gut microbiota interactions, providing new insights into the prevention, diagnosis, and treatment of CRC. EVs can be secreted not only by the host’s own cells and gut microbiota, but also enter the body through the ingestion of exogenous substances, such as probiotics, milk, and plants. EVs from these sources mediate interactions that influence various aspects of CRC development, including modulation of the intestinal barrier, immune responses, and direct effects on tumors. The potential of EVs as biomarkers for CRC is also promising, particularly due to their presence in body fluids such as blood, stool, and urine.

However, the use of EVs in CRC faces several challenges. The diversity of EVs and the lack of standardized protocols for their isolation, enrichment, and characterization remain significant barriers in basic research of EVs. Additionally, the complete mechanisms by which EVs are secreted, altered, and function during CRC development, as well as the translation of findings from cellular and animal models to clinical practice, require further research. Future research should focus on improving methods for the isolation, enrichment, and characterization of EVs, conducting functional analyses of individual EVs, elucidating mechanistic pathways through which EVs affect CRC, establishing comprehensive evaluation criteria for basic research and clinical trials on EVs, and conducting clinical trials to validate the efficacy and safety of EVs in CRC diagnosis and treatment.

In brief, studying EVs in the context of host-microbiota interactions opens new avenues for understanding the pathobiology of CRC. The researches of EVs have the potential to significantly advance the prevention, diagnosis, and treatment of this challenging disease. It is believed that with ongoing research, EVs will play a key role in the future medical diagnosis and treatment of CRC.

## Data Availability

No datasets were generated or analysed during the current study.

## Abbreviations

CRC: Colorectal Cancer
EVs: Extracellular Vesicles
BEVs: Bacterial Extracellular Vesicles
BMVs: Bacterial Membrane Vesicles
OMVs: Outer Membrane Vesicles
miRNAs: microRNAs
